# An analysis of student essays on medical leadership and its educational implications in South Korea

**DOI:** 10.1038/s41598-022-09617-8

**Published:** 2022-04-06

**Authors:** I Re Lee, Hanna Jung, Yewon Lee, Jae Il Shin, Shinki An

**Affiliations:** 1 Department of Medical Education, Yonsei University College of Medicine, Yonsei-ro 50, Seodaemun-gu, CPO Box 8044, Seoul, 03722 Republic of Korea; 2 Eulji University School of Medicine, Daejeon, Republic of Korea; 3 Department of Pediatrics, Yonsei University College of Medicine, Seoul, Republic of Korea; 4 Yonsei Institute for Global Health, Yonsei University Health System, Seoul, Republic of Korea

**Keywords:** Health care, Medical research

## Abstract

To examine medical students’ perceptions of leadership and explore their implications for medical leadership education. We conducted a qualitative analysis of the essays submitted by students in the medical leadership course from 2015 to 2019. We categorised the essays by the characteristics of the selected model leaders (N = 563) and types of leadership (N = 605). A statistically significant proportion of students selected leaders who were of the same gender as themselves (P < 0.001), graduate track students chose leaders in science (P = 0.005), while; military track students chose leaders in the military (P < 0.001). Although the highest proportion of students chose politicians as their model leaders (22.7%), this number decreased over time (P < 0.001), and a wider range of occupational groups were represented between 2015 and 2019. Charismatic leadership was the most frequently selected (31.9%), and over time there was a statistically significant (P = 0.004) increase in the selection of transformational leadership. Students tended to choose individuals whose acts of leadership could be seen and applied. Medical leadership education should account for students’ changing perceptions and present a feasible leadership model, introducing specific examples to illustrate these leadership skills.

## Introduction

Contemporary medical environments are facing complex issues, such as rising costs of treatment and inadequate access to and inconsistent quality of health care^1^. To address the ever-perplexing issues in medicine, there is an increasing need for effective leadership in health care^2,3^. In the past, medical care was primarily conducted by an individual physician. In addition, medical education heavily focused on the diagnosis and treatment of illnesses rather than working as a team to provide solutions that ensure higher quality medical care and safety^4^. However, in modern health care environments, a doctor’s role as a leader has become much more significant not only in physician–patient relationships but also in coordinating team-based tasks in the hospital and managing medical organizations^5^. For instance, as the socioeconomic environment becomes an essential component of a community’s health, physicians are expected to exert leadership in organisations that address public health issues^6^. Accordingly, physicians must be prepared to serve as leaders in health care.

Following the increasing need for leadership in healthcare, leadership skills are being included in physician evaluation criteria. The Association of American Medical Colleges has included leadership as the core requirement for medical students entering residency^7^. The Royal College of Physicians and Surgeons in Canada also includes the role of a *leader* as one of the main capability frameworks and has reflected this in their medical education^8^. Medical schools in the United States are proceeding with various leadership programs and incorporating leadership curricula into their undergraduate medical education^9^. Further, research shows that medical students now recognize the need for leadership education following the changing environment; 85% of medical students agreed that they should be taught leadership communication skills and teamwork abilities during their medical school years^10^. Korean medical educators also attempt to incorporate medical leadership education into medical education curriculum^11^. Yonsei University College of Medicine (YUCM) offers a leadership curriculum, *Doctoring & Medical Humanities: Medical Leadership* (DMH-ML), which is a core course covering 16 h (two hours per week for eight weeks) and offered to first-year medical students in the final quarter since 2014. The first 3 weeks feature lectures on basic concepts of leadership. The next three weeks are divided into three elective tracks, from which students choose lessons about leadership taken from: (1) the history of Severance Hospital in South Korea; (2) medical missions and international public health development; (3) business aspects of medicine. The final two weeks of the curriculum provide a summary of the topics covered. The written assignment of the course is a leadership model critique whereby students select a leader of their choice, summarize the leader’s accomplishments, and analyse the strengths and weaknesses found in that leadership. The course aims to facilitate medical students’ understanding of the nature of leadership from various leaders and help them recognize that their role as a leader is one of the fundamental responsibilities as physicians. All students who participated in the class submitted the written assignment, and the prompts for the written assignments were not changed between 2015 and 2019.

As no profiles have been reported on the leader models selected by medical students to date, in this study, we aimed to examine the medical students’ perceptions of leadership and provide directions for leadership education by analysing the characteristics and types of leadership models presented in leadership model critique essays.

## Results

We analysed a total of 585 essays submitted between 2015 and 2019. After excluding 35 essays that did not present a model, and double-counting 13 essays that presented two individuals, a total of 563 essays were chosen for this study (125 in 2015, 84 in 2016, 113 in 2017, 120 in 2018, and 121 in 2019). Of the 563 essays, 407 (72.3%) were written by male students and 156 (27.7%) by female students. Regarding admission types, 381 students (67.7%) were identified as *undergraduate*, 153 students (27.1%) as *transfer/graduate,* and 29 students (5.2%) as *military* (Table 1). We analysed the demographic characteristics of the model leaders selected in the essays (Table 2). A total of 563 individuals were selected as model leaders, 499 men (88.6%), 55 women (9.8%) and 9 other (1.6%), such as names of industries. The comparison of the gender ratio between the selected model leaders and the students showed that male students tended to select male leaders while female students were significantly more likely to select female leaders (P < 0.001) (Table 3). A total of 331 leaders (58.8%) belonged to the *present generation* category, and 232 (41.2%) belonged to the *previous generation* category. The occupational groups of the model leaders were as follows: politics (n = 128, 22.7%), business (n = 121, 21.5%), science (n = 117, 20.8%), sports (n = 45, 8.0%), social activism (n = 34, 6.0%), arts (n = 33, 5.9%), military (n = 32, 5.7%), religion (n = 18, 3.2%), education/law/exploration (n = 7, 1.2%), and other (n = 28, 5.0%). The comparative analysis of the selected model leaders’ occupational groups and the demographic characteristics of the students showed that a statistically significant proportion of female students (P = 0.0014) chose leaders in science, and a statistically significant proportion of male students chose leaders in sports (P = 0.003) (Table 4). Further, a statistically significant proportion of *undergraduate* students (P = 0.049) chose leaders in politics, *transfer/graduate* students (P = 0.005) chose leaders in science, and *military* students chose leaders in the military. When we analysed the changes in the occupational groups of the selected model leaders from 2015 to 2019, the decrease in the number of students who chose leaders in politics was statistically significant (P < 0.001), and the increase in the number of students who chose leaders in sports was statistically significant (P = 0.015) (Table 5).

**Table 1:** Demographic characteristics of medical students in the leadership curriculum course whose essays were selected for this study.

Characteristic	N (% of 563)
**Gender**	
Male	407 (72.3)
Female	156 (27.7)
**Type of admission**	
Undergraduate students	381 (67.7)
Graduate students	153 (27.1)
Military	29 (5.2)

**Table 2:** Demographic characteristics associated with the selected model leaders in the essays.

Characteristic	N (% of 563)
**Gender**	
Male	499 (88.6)
Female	55 (9.8)
Other	9 (1.6)
**Generation**	
Present	331 (58.8)
Previous	232 (41.2)
**Occupation**	
Politics	128 (22.7)
Business	121 (21.5)
Science	117 (20.8)
Sports	45 (8.0)
Social activism	34 (6.0)
Arts	33 (5.9)
Military	32 (5.7)
Religion	18 (3.2)
Education/law/exploration	7 (1.2)
Other	28 (5.0)

**Table 3:** Comparison of the gender ratio between the selected model leaders and the student who wrote the essays.

	N	Male leaders	Female leaders	P-value
Male students	399	377 (75.6)	22 (40.0)	< 0.001
Female students	155	122 (24.4)	33 (60.0)	
Total	554	499 (100.0)	55 (100.0)	

Data are presented as number (%) unless otherwise indicated.

**Table 4:** Comparison of the selected model leaders’ occupational group and the demographic characteristics of the students who wrote the essays.

	Gender		P-value	Type of admission			P-value
	Male N (% of 407)	Female N (% of 156)		Undergraduate N (% of 381)	Graduate N (% of 153)	Military N (% of 29)	
Politics	94 (73.4)	34 (26.6)	0.742	96 (75.0)	24 (18.8)	8 (6.6)	0.049
Business	89 (73.6)	32 (26.4)	0.726	81 (66.9)	36 (29.8)	4 (3.3)	0.495
Science	74 (63.2)	43 (36.8)	0.014	69 (59.0)	45 (38.5)	3 (2.6)	0.005
Sports	41 (91.1)	4 (8.9)	0.003	36 (80.0)	8 (17.8)	1 (2.2)	0.174
Social activism	23 (67.6)	11 (32.4)	0.532	23 (67.6)	10 (29.4)	1 (2.9)	0.815
Arts	21 (63.6)	12 (36.4)	0.252	24 (72.7)	9 (27.3)	-	0.379
Military	25 (78.1)	7 (21.9)	0.448	18 (56.3)	3 (9.4)	11 (34.4)	< 0.001
Religion	13 (72.2)	5 (27.8)	0.995	11 (61.1)	7 (38.9)	-	0.504
Education/law, exploration	5 (71.4)	2 (28.6)	0.959	2 (28.6)	5 (71.4)	-	0.062
Other	22 (78.6)	6 (21.4)	0.522	21 (75.0)	6 (21.4)	1 (3.6)	0.831

**Table 5:** Changes in the occupational groups of the selected model leaders from 2015 to 2019.

	2015 N (% of 125)	2016 N (% of 84)	2017 N (% of 113)	2018 N (% of 120)	2019 N (% of 121)	P-value
Politics	42 (33.6)	24 (28.6)	23 (20.4)	20 (16.7)	19 (15.7)	< 0.001
Business	25 (20.0)	11 (13.1)	31 (27.4)	25 (20.8)	29 (24.0)	0.259
Science	19 (15.2)	12 (14.3)	34 (30.1)	22 (18.3)	30 (24.8)	0.061
Sports	4 (3.2)	6 (7.1)	9 (8.0)	13 (10.8)	13 (10.7)	0.015
Social activism	8 (6.4)	11 (13.1)	5 (4.4)	4 (3.3)	6 (5.0)	0.122
Arts	10 (8.0)	2 (2.4)	1 (0.9)	14 (11.7)	6 (5.0)	0.771
Military	9 (7.2)	7 (8.3)	4 (3.5)	8 (6.7)	4 (3.3)	0.185
Religion	5 (4.0)	3 (3.6)	3 (2.7)	5 (4.2)	2 (1.7)	0.461
Education, law, exploration	–	1 (1.2)	2 (1.8)	2 (1.7)	2 (1.7)	0.246
Other	3 (2.4)	7 (8.3)	1 (0.9)	7 (5.8)	10 (8.3)	0.096

### Qualitative analysis

We analysed the leadership types of the selected models in 563 essays according to a qualitative framework developed from thematic and content analysis. Based on the analysis, a total of 605 essays were selected (seven essays with no specific category of leadership type were excluded, and 49 essays that presented two types of leadership were counted twice). Six types of leadership were identified in the following order: (1) Charismatic leadership (193; 31.9%) represented by the keywords “authority”, “ability”, “drive”, “firmness”, “determination”, and “strong execution”, (2) Servant leadership (150; 24.8%) by the keywords “sacrifice”, “serving”, “devotion”, “empathy”, “listening”, “respect”, “embrace”, “humility”, and “love”, (3) Collaborative leadership (117;19.3%) by the keywords “communication”, “team”, “cooperation”, “together”, “member”, “network”, and “horizontal”, (4) Transformative leadership (109;18.0%) by the keywords “change”, “innovation”, “creativity”, “novelty”, “pioneering”, “boldness”, “challenge”, and “creation”, (5) Self-leadership (23; 3.8%) by the key phrases “achievement of one’s goals and achievement of tasks”, and (6) Super-leadership (13;2.1%) by key phrases such as “education”, “teaching”, “human resources”, and “making good leaders” (Table 6). A comparison of the proportion of the leadership types in the selected models from 2015 to 2019 revealed that the selection of the transformative leadership type has significantly increased (P = 0.004) (Table 7).

**Table 6:** Keywords and classification by types of model leadership.

Type	Keyword	N (% of 605)
Charismatic leadership	Authority, ability, drive, firmness, determination, strong execution	193 (31.9)
Servant leadership	Sacrifice, serving, devotion, empathy, listening, respect, embrace, humility, love	150 (24.8)
Collaborative leadership	Communication, team, cooperation, together, member, network, horizontal	117 (19.3)
Transformational leadership	Change, innovation, creativity, novelty, pioneering, boldness, challenge, creation	109 (18.0)
Self-leadership	Achievement of one's goals, achievement of tasks	23 (3.8)
Super leadership	Education, teaching, human resources, making good leaders	13 (2.1)

**Table 7:** Changes in the types of model leadership from 2015 to 2019.

	2015 N (% of 136)	2016 N (% of 84)	2017 N (% of 126)	2018 N (% of 125)	2019 N (% of 134)	P-value
Charismatic leadership	54 (39.7)	18 (21.4)	40 (31.7)	40 (32.0)	41 (30.6)	0.345
Servant leadership	40 (29.4)	21 (25.0)	32 (25.4)	28 (22.4)	29 (21.6)	0.119
Collaborative leadership	19 (14.0)	27 (32.1)	22 (17.5)	27 (21.6)	22 (16.4)	0.935
Transformational leadership	17 (12.5)	9 (10.7)	27 (21.4)	23 (18.4)	33 (24.6)	0.004
Self-leadership	4 (2.9)	8 (9.5)	4 (3.2)	3 (2.4)	4 (3.0)	0.351
Super leadership	2 (1.5)	1 (1.2)	1 (0.8)	4 (3.2)	5 (3.7)	0.115

## Discussion

The role models as leaders selected by students differed on the basis of the students’ gender and admission type. Although male leaders were dominant, the proportion of female leaders selected by female students was higher than that selected by male students. The selection of the contemporary leaders of *the present generation* was more common than those leaders of *the previous generation.* A high proportion of the *transfer/graduate* students, many with bachelor’s degrees in the sciences, chose leaders who worked in science fields, and a high proportion of the *military* students chose leaders related to the military. These findings imply that students tend to admire models as leaders among the contemporary figures whose acts of leadership can be observed in real-time as well as models with whom they share more in common, such as gender, academic backgrounds, or occupations, likely because the actions and achievements of such leaders are more understandable and more applicable to their own lives. The educational implication of these findings is the importance of role modelling as well as the influence of the informal, hidden curriculum^12–17^. Just as clinical knowledge and skills can be transmitted formally and informally in clinical situations, leadership in health care can also be transmitted through formal and informal means^18^. Although there are individuals officially designated as leaders in healthcare settings, the presence of individuals influencing other persons in informal ways should be acknowledged. Since individuals can be role models regardless of whether they are officially designated as leaders or whether they have an educational intention, medical educators need to understand the role of informal leadership training^19^. Although many medical schools strive to implement leadership education using various methods^20^, they overlook how informal leadership such as students’ experiences in leading and organizational culture play an important role in developing students' leadership skills^21^. Therefore, medical schools need to develop a faculty development program based on the importance of role modelling, recognizing the fact that role modelling can have both positive and negative effects on medical students^22^. A training program to enhance the leadership abilities of the instructors for better transfer of knowledge to the new generation of students is necessary^23^.

The occupations of leaders chosen by the students changed over the course of the 5 years analysed. At first, many students chose politicians as their model leaders, but the percentage of politicians selected decreased over time, and a wider variety of occupations were represented. This change implies that the students’ perceptions of leadership are shifting and that leaders recognized by society are emerging in various occupational fields. Therefore, medical leadership education and research need to incorporate the interdisciplinary and transdisciplinary approaches to meet continuous social changes^22^. Building a leadership curriculum based on a balanced interdisciplinary approach through the theoretical background in various fields, introducing specific examples of leadership in various areas, and having students reflect on case studies will help students develop various leadership-related competencies^24^.

The types of leadership delineated by the qualitative analysis of the essays showed that the most common type of leadership among the six types was the charismatic type, which is the most traditional leadership type. The traditional figure of a physician with ability, a firm and determined mind, the power to execute, and authority remains the most prominent model as a leader for medical students. As the charismatic leadership type tends to parallel the traditional heroic medical practice led by one-person, medical educators need to emphasize the possible limitations of charismatic leadership in the current health care context, which requires a substantially more team-based approach. As the ratio of students choosing diversified leadership types has gradually increased, it can be considered that the students’ primary concept of medical leadership is changing according to changes in medical society.

The second and third types of leadership stated by students were the servant and collaborative leadership types, which were increasingly recognized as essential in the healthcare field. Earlier, the servant leadership, with its image of dedication to treating patients and contributing to the community^18^, was exemplified as the prominent model for healthcare^25^. The function of collaborative leadership has been increasingly emphasized in the changing medical environment where facilitating successful collaboration within teams and flexibly adapting to changes is becoming more important^26^. Moreover, effective team management and cooperation in health care are known to be closely related to improved outcomes in the treatment of patients^27^. The prevalence of the selection of these types of leadership by the students may reflect their correct understanding of the modern health care approach.

The proportion of transformational leadership increased significantly over time. Transformational leadership is a more suitable leadership type for a constantly changing environment such as that of health care where quick adaptation and decision-making are required^25,28^. Recently, The fourth industrial revolution is characterised by developments such as precision medicine, AI-based medical treatment, and telemedicine, and related discussions are underway in medical education. This increase in the proportion of transformational leadership indicates that students recognize the importance of leadership that is sensitive to change and can respond quickly and with sound judgment.

When we compared the selected leaders' occupations and leadership types, it was confirmed that the students presented various leadership types in the same occupational group (Supplementary Table S1). This finding implied that there is no stereotyped leadership for a specific occupation but that different types of leadership can be manifested depending on the situations and followers in regard to which the leadership is exercised^28^. In other words, physicians as a leader needs to lead organizations, teams, or themselves using various leadership types rather than pursuing one fixed style. Moreover, mature leaders are more proficient in using different types of leadership, and different leadership levels require different skills^29^. These findings suggest that leadership in health care can be learned through case studies of other occupational groups and the curriculum should include various leadership types rather than emphasizing one style.

### Limitations

This study has the following limitations. First, the sample of this study is limited to the medical students in South Korea. Considering that effective leadership behaviors are being accepted to be culture-specific, it is difficult to generalize the qualitative analysis conducted on essays collected from a single medical school^30^. Second, although the percentage of students in each admission type corresponds with the average percentages of undergraduate track (70%) and graduate track (30%) admissions in South Korea, the fact that students in the graduate track would have been in their first year of medical school at the time of essay submission is a limitation. Third, it is possible that the essays submitted by the students were influenced by the lectures held in class. In selecting a model leader, the student may have considered leaders, leadership theories, and types of leadership presented by the instructor. Nevertheless, this study is meaningful because it explores the experiences of the medical students over the past five years, analyses leadership recognized by the students, and examines the changes in their perceptions over time.

## Conclusions

Whether leadership is innate or acquired remains a matter of debate, but many experts argue that education and experience can teach the skills and behaviours necessary for developing the ability to lead others^23,31^. Therefore, a well-designed leadership curriculum that presents feasible leadership models is needed because students imitate familiar and applicable leaders. Further, in the rapidly changing medical environment, leadership roles are diversifying, and students' perceptions of leadership are changing. Therefore, when medical schools encourage the various approaches to leadership required in modern society, students can foster broad skills in medical leadership.

## Methods

**Figure 1:**
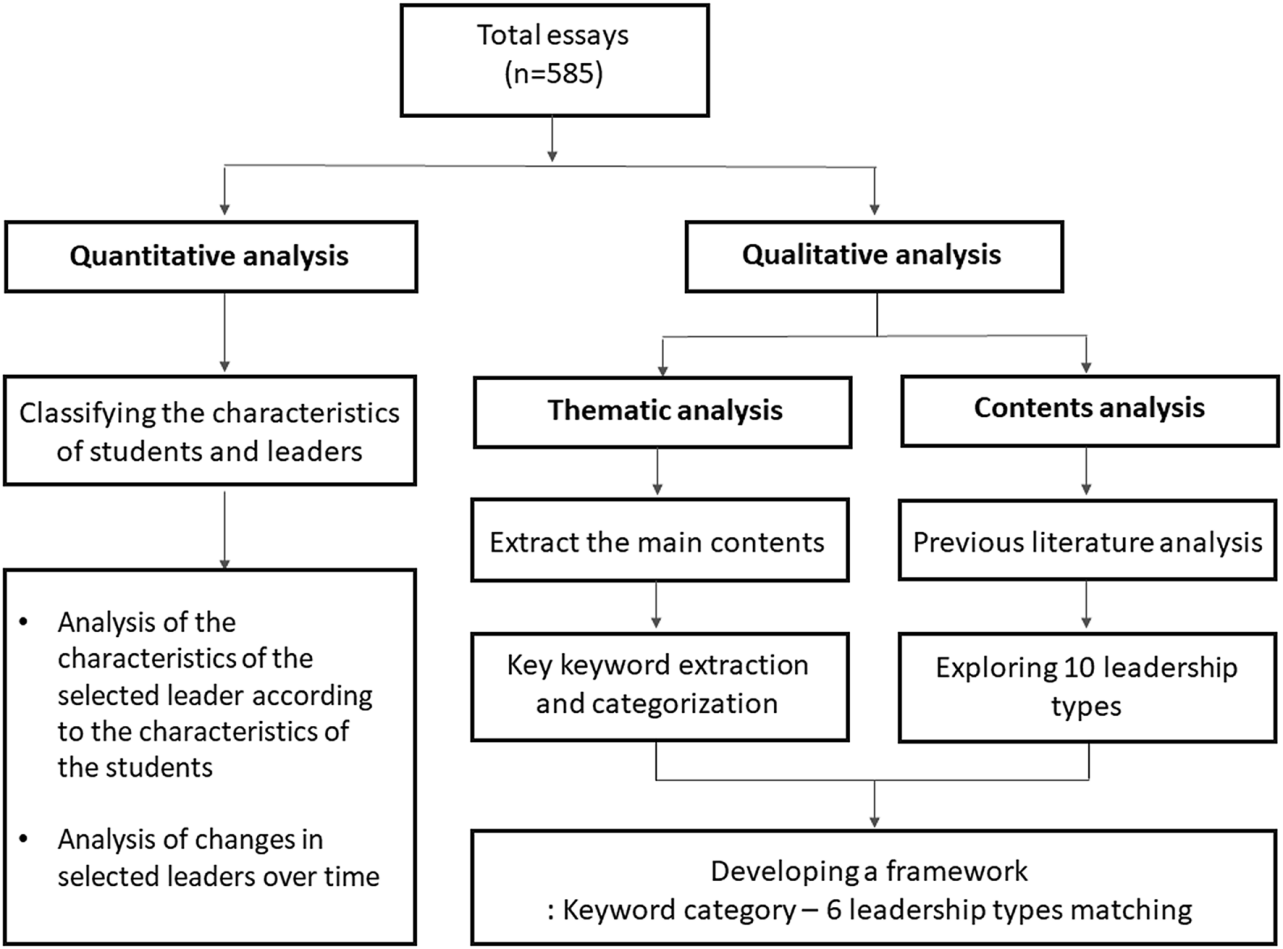
Schematic diagram of quantitative and qualitative analyses on the essays.

We reviewed all essays submitted in the first-year core course, titled *Doctoring & Medical Humanities: Medical Leadership*, from 2015 to 2019, to investigate changes in the perceptions of leadership among medical students. The prompt of the essay required students enrolled in the DMH-ML course to select a model leader, summarize that leader’s achievements, and reflect on the strengths and weaknesses of leadership found. We collected a total of 585 essays and performed quantitative and qualitative analysis (Fig. 1).

### Student demographics and data collection

**Figure 2:**
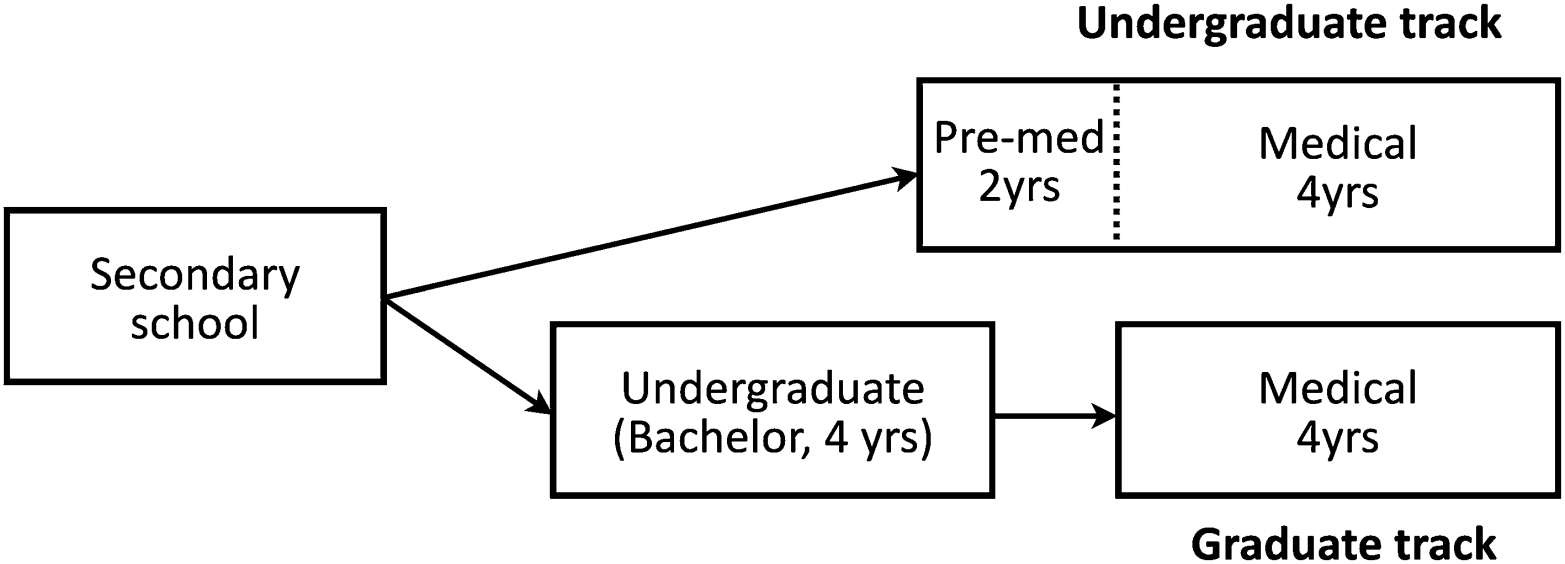
Schematic diagram of medical educational system in South Korea.

To perform quantitative analysis, we classified the characteristics of the students as well as those of the leaders they selected. We collected demographic information such as gender and type of admission of the medical students at YUCM who submitted the essays and classified them into three groups: (1) undergraduate track, (2) graduate track, and (3) military track. The *undergraduate* track is a conventional 6-year program in South Korea and is for students who have immediately graduated from high school. The first two years are equivalent to the *pre-med* years of an undergraduate degree, and the remaining 4 years are equivalent to the *medical* years (2 years for preclinical and 2 years for clerkship) of medical schools elsewhere. Thus, by the time of their essay submission, students in the undergraduate track would be in their third year having enrolled at medical school. The *graduate* track is a four-year program for those with an undergraduate degree. Thus, students transfer straight into the medical years, skipping the pre-med years of medical school. This track is typical of admission to medical school in the United States and Canada. In Australia, England, Ireland, Singapore and South Korea, the *undergraduate* track and the *graduate* track are mixed (Fig. 2)^32^. Finally, the *military* track is for the military students with an undergraduate degree commissioned by the army.

### Quantitative analysis

We also classified the gender, generation, and occupational groups of the selected model leaders. We classified the selected leaders as (1) the *previous generation* if they had passed away before 2000 and (2) the *present generation* if they had passed away after 2000 or were still living at the time of the study. The occupational groups of the model leaders were classified as politics, business, science, sports, social activism, arts, military, religion, and education/law/exploration. In addition, when students selected an individual with whom they had a personal relationship such as a parent or a character in a book or movie, we classified them as “other”.

After classifying the characteristics of students and leaders, we analysed the characteristics of selected leaders according to the characteristics of students and observed how the students' perceptions of leadership changed over time from 2015 to 2019.

### Qualitative analysis

We used a combination of thematic and contents analyses for our qualitative analysis^33,34^. Two authors independently analysed each essay. We omitted essays that did not establish a model leader. For essays with two selected leaders, we analysed them as two separate model leaders. The strengths of each selected model leader portrayed by students were summarized. Disagreements were resolved through group discussion and consensus.

In the first step, we extracted the main contents that delineated the selected leaders' performance, strengths, and weaknesses from the essays for thematic analysis. We then, classified these extracted contents by thematic keywords with similar meanings.

Second, we developed a framework for content analysis through a review of previously published literature.

Finally, the result of the thematic analysis was combined with the result of the content analysis. The framework was formed based on six types of model leadership by matching the 10 leadership types (adaptive, authentic, charismatic, collaborative, servant, self, situational, super, transformational, and transactional) selected through the analysis of previous studies with the leadership types described by the students^27,35–41^: charismatic, servant, collaborative, transformational, super-, and self-leadership.

The six leadership model types are defined as follows. Charismatic leadership centres on the leader’s strong charisma and resolute style that allows members to follow the decisions they make^35^. Servant leadership is based on respect for humans, whereby the leader volunteers to serve each member to help develop their full potential^36^. Collaborative leadership is exerted by leaders who establish a horizontal and trusting relationship with members that enables the group to complete the given tasks through cooperation^27^. Transformational leadership recognises the need for a change within the organisation and opportunities for a leader to envision and enact change^37^. Self-leadership is a force that drives leaders themselves to accomplish their goals, whereas super-leadership nurtures other individuals(followers) and empowers them to lead themselves^38^.

### Statistical analysis

We used descriptive statistics to analyse the characteristics of the study subjects. We indicated frequencies and percentages for categorical variables, and a chi-square test and linear-by-linear association were performed to analyse the correlation between two categorical variables. Fisher's exact test was performed if the expected frequency was five or less in the chi-square test. All statistical analyses were performed using IBM SPSS ver. 25.0 (IBM Corp., Armonk, NY, USA), and the statistical significance level was set to p = 0.05.

### Ethical considerations

The Yonsei University Health System Institutional Review Board (IRB No: Y-2020-0206) approved the study. We used anonymised materials collected in commonly accepted educational settings according to Article 2 of the Bioethics and Safety Act Enforcement Rule in South Korea. The informed consent requirement was exempt from institutional review board approval. All procedures were conducted in accordance with the relevant guidelines and regulations.

## Supplementary Information

Supplementary Table S1.

## Data Availability

The datasets generated during and/or analysed during the current study are available from the corresponding author on reasonable request.
