# Supplementary material for: An analysis of student essays on medical leadership and its educational implications in South Korea
Source: Sci Rep. 2022 Apr 6;12:5788. doi: 10.1038/s41598-022-09617-8 (PMC8987100; doi:10.1038/s41598-022-09617-8)
Supplement: Supplementary file 1 — Supplementary Table S1. [file 41598_2022_9617_MOESM1_ESM.docx]

**An analysis of student essays on medical leadership and its educational implications in South Korea**

I Re Lee, Hanna Jung, Yewon Lee, Jae Il Shin, Shinki An

Supplementary Table S1. Comparison of the selected role models’ occupational groups and the types of model leadership

|  | **Charismatic** | **Servant** | **Collaborative** | **Transformational** | **Self-** | **Super-** | **Total**  N (%) |
| --- | --- | --- | --- | --- | --- | --- | --- |
| **Politics** | 72 (51.8) | 25 (18.0) | 21 (15.1) | 19 (13.7) | 2 (1.4) | - | 139 (100.0) |
| **Business** | 48 (37.2) | 6 (4.7) | 28 (21.7) | 44 (34.1) | 1 (0.8) | 2 (1.6) | 129 (100.0) |
| **Science** | 22 (17.1) | 50 (38.8) | 16 (12.4) | 30 (23.3) | 7 (5.4) | 4 (3.1) | 129 (100.0) |
| **Sports** | 16 (34.8) | 9 (19.6) | 15 (32.6) | 1 (2.2) | 4 (8.7) | 1 (2.2) | 46 (100.0) |
| **Social activism** | 9 (24.3) | 17 (45.9) | 4 (10.8) | 4 (10.8) | 2 (5.4) | 1 (2.7) | 37 (100.0) |
| **Arts** | 6 (17.6) | 9 (26.5) | 7 (20.6) | 7 (20.6) | 4 (11.8) | 1 (2.9) | 34 (100.0) |
| **Military** | 10 (25.6) | 12 (30.8) | 17 (43.6) | - | - | - | 39 (100.0) |
| **Religion** | 2 (11.1) | 13 (72.2) | - | - | 1 (5.6) | 2 (11.1) | 18 (100.0) |
| **Education/**  **Law/**  **Exploration** | 1 (14.3) | 1 (14.3) | 1 (14.3) | 2 (28.6) | 1 (14.3) | 1 (14.3) | 7 (100.0) |
| **Other** | 7 (25.9) | 8 (29.6) | 8 (29.6) | 2 (7.4) | 1 (3.7) | 1 (3.7) | 27 (100.0) |
